# Change in diet, physical activity, and body weight among young-adults during the transition from high school to college

**DOI:** 10.1186/1475-2891-8-32

**Published:** 2009-07-22

**Authors:** Heidi J Wengreen, Cara Moncur

**Affiliations:** 1 Department of Nutrition and Food Sciences, Utah State University, Logan, Utah, USA

## Abstract

**Background:**

The freshmen year of college is likely a critical period for risk of weight gain among young-adults.

**Methods:**

A longitudinal observational study was conducted to examine changes in weight, dietary intake, and other health-related behaviors among first-year college students (n = 186) attending a public University in the western United States. Weight was measured at the beginning and end of fall semester (August – December 2005). Participants completed surveys about dietary intake, physical activity and other health-related behaviors during the last six months of high school (January – June 2005) in August 2005 and during their first semester of college (August – December 2005) in December 2005.

**Results:**

159 students (n = 102 women, 57 men) completed both assessments. The average BMI at the baseline assessment was 23.0 (standard deviation (SD) 3.8). Although the average amount of weight gained during the 15-week study was modest (1.5 kg), 23% of participants gained ≥ 5% of their baseline body weight. Average weight gain among those who gained ≥ 5% of baseline body weight was 4.5 kg. Those who gained ≥ 5% of body weight reported less physical activity during college than high school, were more likely to eat breakfast, and slept more than were those who did not gain ≥ 5% of body weight.

**Conclusion:**

Almost one quarter of students gained a significant amount of weight during their first semester of college. This research provides further support for the implementation of education or other strategies aimed at helping young-adults entering college to achieve or maintain a healthy body weight.

## Background

The high prevalence of obesity in modern societies is a major public health threat and contributes to preventable morbidity and mortality. Current obesity rates among all age groups are two-to-three times higher than they were just 20 years ago [1]. According to the Behavioral Risk Factor Surveillance System (BRFSS; 1991–1998), the greatest increases in obesity rates were among 18–29-year-olds and those who had some college education [2,3]. A recent report from the American College Health Association [2] reported 36.7% of college students were overweight or obese based on self-reported height and weight values.

The phenomenon of gaining weight during a person's first year of college is familiar to most college students. Several research groups have examined this phenomenon and most, [4] but not all, [5] reported weight changes among students during their first year of college. Average weight gain during the first semester of college for first-time freshmen was 1.3 – 3.1 kg [6-10].

The transition from high school to college often results in drastic changes to environment and resources, and such changes likely impact health-related behaviors [11-13]. Many studies have documented unhealthy behaviors among college students including decreased physical activity, increased rates of smoking and drinking, and decreased overall diet quality yet few studies have examined the change in behaviors that occur as students' transition from high-school to college [4,12-14]. The objective of this study was to examine associations between changes in diet, physical activity, weight, and body mass index (BMI) among 18–19-year-olds during their transition to college life. It is hypothesized that weight gain is common during the first semester of college and is associated with change in behaviors that impact energy balance, including diet and physical activity.

## Methods

The Freshmen Health Study (FHS) was a longitudinal study of 186 college freshmen (68 men, 118 women) that examined changes in diet, physical activity and other health-related behaviors, and weight during their first semester of college. This study took place at a mid-sized land-grant institution located in the western region of the United States. First-year college students who attended high school during the previous year, who were not pregnant, and were 18–19 years of age were eligible to participate. Participants were recruited using convenience-sampling methods at various venues on campus during the two weeks prior to the beginning of fall semester 2005 when a freshmen orientation course was being held. Of the 2,054 first-time freshmen enrolled at the University in 2005, 1,388 participated in the freshmen orientation course, 200 of which were recruited to participate in the "Freshmen Health Study". The exclusion criteria were listed on recruitment posters and fliers; therefore no students were excluded after enrolment. The goal of recruiting 200 students was determined by the resources available to conduct the study. Of the 200 students who were recruited to participate, 186 (93%) completed the first assessment (August 2005), and 159 (80%) completed the second assessment (December 2005).

Participants were assessed during two data collection periods: first during the last two weeks of August 2005 and again during the first two weeks of December 2005. For 95% of the sample, the second assessment was conducted during the week prior to final exams in December. Participants received a T-shirt and ten dollars per data collection as compensation. All methods and procedures were reviewed and approved by the institute's Institutional Review Board, and all participants signed an informed consent prior to participating.

At each of the two data collections participants were weighed, measured, and asked to complete a survey that included questions about diet, physical activity, personal and family history of health, as well as other health-related behaviors. The baseline survey asked participants to retrospectively report about their behaviors during their last six months of high school (January – May 2005). The follow-up survey asked participants to report about those same behaviors during fall semester (August – December 2005). Differences in the two reports represented change in behavior that occurred during the transition from high school to college.

### Assessment of weight change

Trained research assistants measured participants in light clothing without shoes, jackets, or heavy items in their pockets. Weight to the nearest tenth of a kilogram (kg) was measured on a Taylor PrecisionTECH calibrated digital scale (Taylor Precision Products, Oak Brook, IL). Height was measured to the nearest centimetre using a portable Ross Stadiometer (Ross Laboratories, Columbus, OH). Body mass index (BMI) was calculated from measured height and weight (kg/m2). Adult BMI criteria was used to categorize participants as overweight (BMI ≥ 25) or not-overweight (BMI <25) [15]. To account for variability in the absolute amount of weight change by differences in body mass, percent change in body weight was determined and a change in weight greater than or equal to five percent (≥ 5%) of baseline body weight was defined as a clinically significant change [16,17].

### Assessment of exposure variables

A self-administered modified version of the Food Frequency Questionnaire (FFQ) previously validated for use in a general population of adults 20 years of age and older and used in the Nurses' Health Study [18] was used to estimate usual dietary intake. The questionnaire was modified to include a food item for sports and energy drinks and sports and energy bars. Using this method, participants were asked to report their usual frequency of consumption of a list of 142 food items over the specified time periods. The time period for the assessment collected in August was the last six months of high school. The time period for the assessment collected at the end of fall semester (November – December) was the last four months. Following the methods used in the Nurses' Health Study, foods were presented in standard serving sizes. The FFQ included 16 dairy foods, 17 fruits, 28 vegetables, 22 meat dishes, 18 bread, grains and cereals, 17 beverages other than milk, and 24 snack-type foods. Nutrient intake was calculated by multiplying the frequency of consumption of the specified food item or group by the nutrient content of that food; total nutrient intake was obtained by summing across all foods. The ESHA nutrient composition database (Food Processor ESHA Research version 10), which is based primarily on USDA's National Nutrient Database for Standard Reference but also includes nutrient content information from manufacturers, was used to assign nutrient content of foods. Intake of carbohydrate, protein, and fat are expressed as a percent of total caloric intake.

Physical activity (PA) was assessed by asking participants to report how often (days per week) they participated in moderate or vigorous activities (such as brisk walking, jogging, biking, aerobics, or yard work), in addition to their normal daily routine, a method modelled after USDA's MyPyramid classifications for placing individuals into broad categories based on energy expenditure [19]. At the second interview participants were also asked if they participated in more, the same, or less PA than the amount they participated in during the last six-months of high school.

In addition, participants were asked to report about their self-perceived health (less or more healthy than peers) and health behaviors that included use of dietary supplements, breakfast consumption, alcohol use and smoking, and number of meals eaten per week at convenience or fast-food type dining establishments and on-campus dining facilities. Participants also categorized their parents as being currently underweight, of a healthy weight, overweight, or obese. The questions included in this survey were similar to questions included in surveys of previously published studies but has not been previously validated.

### Statistical Analysis

All statistical analyses were performed using SPSS software version 15.0. Means and standard deviations were used to describe the distribution of continuous variables. Analysis of variance (ANOVA), an extension of the two-sample t test, and Chi-squared distributions were used to compare means and percentages across weight status and weight gain groups. A repeated-measures ANOVA was used to examine the difference in mean height, weight, and BMI measured in August 2005 and again in December 2005. All statistical tests conducted were two-sided with a type I error (α) of 0.05, and P values < 0.05 were considered statistically significant.

## Results

Of the 185 participants who were weighed and measured during August of 2005, 4% (n = 8) had a BMI of < 18.5, 70% (n = 139) had a BMI of 18.5 to 24.99, 14% (n = 27) had a BMI of 25 to 29.99, and 6% (n = 11) had a BMI of 30 or greater. More women (63%) than men (37%) chose to participate in the study although gender was neither associated with prevalence of a BMI ≥ 25 upon entering college (p = 0.146) nor risk of weight gain during the first semester of college (p = 0.251). Ninety-seven percent reported their ethnicity as non-Hispanic white, a percent slightly higher than the 91.1% of freshmen enrolled at the university who reported their ethnicity as non-Hispanic white. Participants who entered the study with a BMI ≥ 25 were more likely to drop out of the study than were those with an initial BMI < 25. One hundred fifty-nine (85% of the original cohort) participants provided follow-up data (n = 102 females, 57 males).

Characteristics of the study population by BMI status are presented in table 1. There were few statistically significant differences in characteristics related to diet, physical activity or other health-related behaviors between baseline BMI groups. Those with a BMI of ≥ 25 reported being less healthy than their peers and were more likely to be unhappy with their weight than were those with a BMI < 25 (p-value 0.003, < 0.001, respectively). In addition, those with a BMI of ≥ 25 were more likely to report their mothers, but not their fathers, as being overweight than were those with a BMI of < 25 (p-value 0.006, 0.146, respectively).

**Table 1:** Characteristics of the Freshmen Health Study participants by BMI status (BMI < 25; BMI ≥ 25) upon entering college (2005).

Characteristic	Baseline BMI < 25 (n = 131)	Baseline BMI ≥ 25 (n = 28)	p-value
White-non-Hispanic (%)	97%	95%	0.746

Female (%)	66.2	52.6	0.121
BMI (kg/m2)	21.5 ± 2.0	28.9 ± 3.8	<0.001
Happy with current weight^b^(%)	60.8	27.8	<0.001
Reported self as less healthy than peers (%)	2.1	13.5	0.003
Ate breakfast regularly (at least 4 times per week)^b^(%)	72.1	64.9	0.387
Ever drank alcohol^b^(%)	12.7	24.3	0.154
Ever smoked cigarettes^b^(%)	6.3	5.4	0.856
Participated in vigorous physical activity most days of the week^b ^(%)	44.2	45.2	0.320
Participated in organized sports during High School (%)	51.6	43.8	0.427
Reported mother being overweight/obese (%)	53.0	77.8	0.002
Reported father being overweight/obese (%)	52.0	68.6	0.146
Dietary intake as estimated by baseline FFQ^b, c^			
Calories per day	2320 ± 1021	2363 ± 776	0.86
Energy as a percent of total calories			
Protein	16.17 ± 3.0	16.33 ± 3.5	0.72
Carbohydrate	51.59 ± 6.9	52.54 ± 6.8	0.45
Fat	34.07 ± 5.9	32.87 ± 5.1	0.23
Servings (.5 cup) of fruit per day	2.22 ± 1.69	2.65 ± 1.74	0.179
Servings (0.5 cup) of vegetables per day	3.41 ± 4.18	3.53 ± 2.20	0.865
Sweetened-carbonated beverages per day	0.64 ± 0.98	0.85 ± 1.05	0.268
Servings (1 cup) of milk per day	1.97 ± 1.65	1.90 ± 1.29	0.820
Carbonated beverages per day	0.87 ± 1.43	1.16 ± 1.43	0.283
Occasions when junk food was eaten as a snack per day	0.99 ± 0.95	0.84 ± 0.96	0.385
Fast food meals per week	2.22 ± 2.5	2.23 ± 2.4	0.920

^a^159 students were weighed and measured at the second data collection period including 131 with a baseline BMI of <25 and 28 with a baseline BMI of ≥ 25.

^b^Dietary and lifestyle habits are for the period of time January 2005 – June 2005, or the last six months of high school.

^c^14 participants were excluded do to implausible energy intakes of <500 calories or >6,000 calories.

Despite no significant change in height (p-value = 0.615), both weight and BMI increased from August to December (p-value = < 0.001 for both). Average weight change during this time was 1.51 (± 2.3) kg; there was no significant difference in the amount of weight gained by men and women (p-value for the effect of gender on weight change = 0.235). Because BMI is a ratio of height for weight and men were on average taller than women, average change in BMI was different for men and women (p-value for the effect of gender on change in BMI = 0.048); men increased in BMI by an average 0.33 (± 0.84) points and women increased in BMI by an average 0.60 (± 0.77) points.

Seventy-seven percent (n = 123) of participants maintained their body weight to within 5% of their baseline body weight. Twenty three percent of participants (n = 36) gained ≥ 5% of their body weight during the approximately 16-week period between August and December; no participant lost ≥ 5% of body weight during the same period. The cut-off of ≥ 5% of body weight represented the 78% percentile for the distribution of weight change among study participants. Among those who gained ≥ 5% of body weight, the average amount of weight gained was 4.5 kg (9.9 lbs) and nine converted from a BMI < 25 to a BMI ≥ 25. The total percent of participants with BMI ≥ 25 increased from 20% at the beginning of fall semester to 23% at the end of fall semester (paired t-test p-value = 0.004).

Characteristics of the study population by weight gain status during fall semester are presented in table 2. Participants who gained ≥ 5% of body weight (n = 36) were more likely to eat breakfast, reported a greater amount of average sleep, and participated in less physical activity during their first three months of college compared to the amount they participated in during high school than did those who did not gain ≥ 5% of body weight (p-values = 0.05, 0.006, 0.05, respectively). After adjusting for the problem of multiple comparisons by making a Bonferroni correction to the level of p-values considered statistically significant, only the association between average sleep and significant weight gain remained statistically significant with a type I error (α) of 0.05. Other characteristics and behaviors were similar between the two groups.

**Table 2:** Characteristics of the Freshmen Health Study participants in December of 2005 by weight change status.

Characteristic	No significant weight gain (n = 123)	Weight gain of ≥ 5% of body weight (n = 36)	p-value
Female (%)	61.8	72.2	0.25
Baseline BMI (kg/m2)	23.33 ± 3.80	21.41 ± 2.8	0.005
BMI at end of fall semester	23.53 ± 3.69	22.95 ± 3.0	0.39
Weight change during fall semester (kg)	0.63 ± 1.57	4.52 ± 1.61	<0.001^c^
Height change during fall semester (cm)	0.08 ± 0.84	0.07 ± 0.67	0.90
Ate breakfast regularly^b ^(%)	65.0	86.1	0.05
Ever drank alcohol^b ^(%)	10.6	13.9	0.58
Ever smoked cigarettes^b ^(%)	2.4	2.8	0.91
Participated in vigorous exercise most days of the week^b ^(%)	23.7	12.5	0.39
Participate in less physical activity in college as compare to high school (%)	43.6	60.7	0.05
Lived on campus (%)	63.0	79.4	0.07
Dietary intake as estimated by wave 2 FFQ^a, b^			
Calories per day	1693 ± 598	1779 ± 788	0.49
Energy as a percent of total calories			
Protein	15.80 ± 3.11	15.25 ± 3.2	0.36
Carbohydrate	53.17 ± 7.69	52.68 ± 8.7	0.75
Fat	32.79 ± 6.21	33.96 ± 6.9	0.34
Servings of fruit per day	1.63 ± 1.13	1.42 ± 0.98	0.34
Servings of vegetables per day	3.53 ± 1.6	2.94 ± 1.67	0.07
Sweetened-carbonated beverages per day	0.46 ± 0.93	0.53 ± 0.78	0.66
Servings of milk per day	1.58 ± 0.41	1.41 ± 1.14	0.50
Occasions when junk food was eaten as a snack per day	0.91 ± 1.0	1.11 ± 1.12	0.30
Dining-hall meals eaten per week	3.3 ± 5.3	5.4 ± 7.0	0.059
Fast food meals eaten per week	1.35 ± 2.17	1.69 ± 2.19	0.40
Hours of sleep per night	8.65 ± 1.12	7.38 ± 2.24	0.006^c^

^a^9 participants were excluded due to implausible caloric intake of <500 and >6,000 calories per day.

^b^Dietary and lifestyle habits reported during fall semester (August – December).

^c^Remained statistically significant at the type I error (α) of 0.05 after a Bonferroni correction for multiple comparisons.

## Discussion

In this longitudinal study of measured weights and self-reported diet and health behaviors among 159 18-year-old men and women enrolled as freshmen at a mid-sized land grant University in the Western U.S., 20.4% entered college overweight or obese as indicated by a BMI ≥ 25. The average amount of weight gain experienced by these students during their first semester of college was modest (1.5 kg; 3.3 lbs), although 23% of participants gained an amount of weight ≥ 5% of their baseline body weight. The average amount of weight gained among those who gained ≥ 5% of their baseline body weight was 4.52 kg (9.9 lbs).

Like others studying the phenomenon of weight gain among freshmen, we observed that some but not all freshmen do gain a significant amount of weight during their first semester of college. Average weight gain in our study among all participants was 1.5 (SD 2.28) kg, an amount similar to the amount reported in other studies of weight gain among freshmen [4,7,20-22]. Clinically significant weight gain was defined as an amount ≥ 5% of each individual's baseline body weight, a method not previously used in similar studies. This method of defining what is considered clinically significant weight gain may help to control for the large differences in the magnitude of weight gain experienced by men and women due to differences in body mass. The average amount of weight gain observed among men and women who gained ≥ 5% of body weight was 5.3 (± 1.9) among men, and 4.2 (± 1.4) kg among women.

In general, our findings are consistent with the findings of others who report the transition from high school to college promotes changes in behavior and environment that may support weight gain [23,24]. Others have identified eating in dining halls as a significant risk factor for weight gain during the first semester of college [7]. Levitsky [7] hypothesized that the greater abundance and variety of food available in dining halls may promote intake in excess of energy needs.

In a community-controlled analysis of weight gain among university women, Hovel et al. [8] found that by the junior year of college average weight returned to near baseline levels of the cohort as entering freshmen. The weight loss among women during their junior year was speculated to be associated with a move from mandatory dormitory housing and dining-hall-type dining experiences to other options. In a study examining weight change across years of college among a small cohort of men and women Racette et al. [25] found that the accelerated rate of weight gain experienced during the freshmen year of college did not continue through senior year and that by senior year most students (85%) had moved from residence halls to off-campus housing. Approximately 65% (n = 102) of the participants in this study lived on campus and reported eating at least occasionally at all-you-can-eat dining facilities. In the present study, those who gained ≥ 5% of body weight ate an average of 2.1 more meals per week in on-campus dining facilities during fall semester (August – December) than did those who did not gain ≥ 5% of body weight, although the statistical significance of this difference is marginal (p-value = 0.06).

Somewhat surprising were the associations between more frequent breakfast consumption and greater amounts of sleep reported among those who gained ≥ 5% of body weight. These associations have not been previously reported in the literature regarding risk factors for weight changes during the transition to college. A substantial body of literature provides evidence that breakfast skipping in children, adolescents, and adults is associated with body weight [26-28]. In this study of first-time freshmen, regular breakfast consumption was marginally associated with on-campus living (p = 0.057); on-campus living was associated with more frequent meals eaten in all-you-can-eat dining facilities (p-value = 0.009). The observed findings of a positive association between breakfast consumption and weight gain may reflect differences in access to all-you-can-eat dining facilities among college freshmen and nationally representative samples of adolescents and adults. The frequency of breakfast consumption in all-you-can-eat dining facilities was not quantified in the present study. In addition, regular breakfast consumption was defined as eating breakfast at least four times per week; additional details about the frequency and type of breakfast consumed may have helped to clarify the observed associations.

Short sleep duration has been associated with obesity in paediatric populations but the existing evidence regarding associations between habitual sleep duration and body weight among adults is not consistent [29]. Our findings regarding associations between sleep patterns and risk for weight gain among college freshmen are novel. Relationships between sleep patterns among young-adults attending college and weight change and other indicators of health are important and deserves further study.

Our study has several limitations. First, participants with a BMI ≥ 25 at the baseline interview were more likely to drop out of the study than were those with BMI < 25. This may have biased our results to the null if those who dropped out were also those who gained weight; those who dropped out may have differed in other important ways from those who continued in the study. For example, participants who began the study with a BMI >25 had higher rates of drinking than did those with BMIs < 25. This may have contributed to differences in the prevalence of alcohol consumption at the August and December assessments.

Second, we assessed diet and physical activity using instruments that relied on the accurate memory recall of usual behaviors by participants. The baseline survey instruments asked participants to report their behaviors during a six-month period of time that occurred three to nine months prior to when the data were collected so as to capture usual behaviors during the period of time that included their last six months of high school. The first FFQ, being more retrospective than the second, may have provided a less accurate estimate of usual dietary intake and physical activity than did the more immediate assessment of behaviors collected at the second data collection period as it is known that reports of past behavior are influenced by current behavior [30]. However, Maruti et al. [31] found that FFQs may be used to provide a reasonable estimate of usual dietary intake in the distant past. In the Maruti et al. [31] study young adults were able to use an FFQ to accurately report usual dietary intake from approximately 10 years in the past using a FFQ.

In addition, although total energy intake between the first and second assessments was correlated (r = 0.57, p-value < 0.001), there were significant differences between the absolute total energy intake reported at the first and second assessments that in general would not support weight gain among the population. Butler et al[4], and Jung et al[9] also found that energy intake decreased significantly during the first semester of college, despite overall increases in weight. Our finding of an association between being less physically active and weight gain during the first semester of college is consistent with the findings of both Butler et al[4], and Jung et al[9] who also found decreased physical activity associated with increased risk for weight gain despite overall decreases in energy consumption.

Third, because we did not assess body composition, we cannot determine whether the observed increases in body weight were associated with growth or increases in lean or non-lean body mass. However, averaged measured heights were not different between the baseline and follow-up assessments, indicating little change in stature during the 16-week study period among our participants.

Finally, the participants in this study were recruited from among first-time freshmen attending one university with a population of students who are predominately non-Hispance white (91%) and who report lower rates of smoking and drinking than reported nationally. This study population likely does not represent the diversity of first-time freshmen attending college campuses nationally.

## Conclusion

The results of this study demonstrate that some, but not all college students experience a significant amount of weight gain during the transition from high school to college. Several factors are certainly involved. This study provides further evidence that the transition to college life is a critical period of risk for weight gain and college freshmen are an important target population for obesity prevention strategies. Targeted information about maintaining energy balance through regular physical activity and appropriate energy intake from a healthy balanced diet, delivered to students at the outset of their college career, may be effective in preventing weight gain among college freshmen during this critical period. Venues for such targeted education may include freshmen orientation sessions, residence halls, and point-of-purchase education in dining facilities.

## Competing interests

The authors declare that they have no competing interests.

## Authors' contributions

HJW and CM made substantial contribution to the conception, design, and acquisition of data. HJW performed the data analysis and interpretation. HJW and CM were involved in drafting the manuscript and have read and approved the final manuscript.
